# Surgery in Recurrent Ovarian Cancer: A Meta-Analysis

**DOI:** 10.3390/cancers15133470

**Published:** 2023-07-02

**Authors:** Maria Teresa Climent, Anna Serra, Maria Llueca, Antoni Llueca

**Affiliations:** 1 Multidisciplinary Unit of Abdominopelvic Oncology Surgery (MUAPOS), Department of Obstetrics and Gynaecology, University General Hospital of Castellon, 12004 Castellon, Spain; serraa@uji.es (A.S.); llueca@uji.es (A.L.); 2 Oncological Surgery Research Group (OSRG), Department of Medicine, University Jaume I (UJI), 12004 Castellon, Spain; 3 Department of Medicine, University CEU-Cardenal Herrera, 12006 Castellon, Spain; mariabenicassim@hotmail.com

**Keywords:** secondary cytoreductive surgery, recurrent epithelial ovarian cancer, overall survival, disease-free survival, systematic revision, meta-analysis

## Abstract

**Simple Summary:**

Ovarian cancer has the highest mortality rate of any type of gynecological cancer because it is diagnosed in advanced stages and its recurrence rate is about 80%. The standard treatment for recurrences of ovarian cancer is systemic chemotherapy. Secondary cytoreductive surgery may be a treatment option for selected patients. We evaluated three randomized studies to determine the effect on overall survival and disease-free survival. This analysis shows better results in this group than in the patients who were treated with chemotherapy alone, with statistically significant differences. This benefit is maintained when analyzing patients in whom complete cytoreduction is achieved. The main limitation of the selected studies is the different criteria for selecting patients for secondary cytoreduction, which is why prospective studies are needed to determine which patients will benefit from this treatment.

**Abstract:**

**Background**: The second cytoreductive surgery performed for a patient who has recurrent ovarian cancer remains controversial. Our study analyzes overall survival (OS) and disease-free survival (DFS) for cytoreductive surgery in addition to chemotherapy in recurrent ovarian cancer instead of chemotherapy alone. **Methods**: A meta-analysis was conducted using PubMed and the Cochrane database of systematic reviews to select randomized controlled studies. In total, three randomized studies were used, employing a total of 1249 patients. **Results**: The results of our meta-analysis of these randomized controlled trials identified significant differences in OS (HR = 0.83, IC 95% 0.70–0.99, *p* < 0.04) and DFS (HR = 0.63, IC 95% 0.55–0.72, *p* < 0.000001). A subgroup analysis comparing complete cytoreductive surgery and surgery with residual tumor achieved better results for both OS (HR = 0.65, IC 95% 0.49–0.86, *p* = 0.002) and DFS (HR = 0.67, IC 95% 0.53–0.82, *p* = 0.0008), with statistical significance. **Conclusions**: A complete secondary cytoreductive surgery (SCS) in recurrent ovarian cancer (ROC) demonstrates an improvement in the OS and DFS, and this benefit is most evident in cases where complete cytoreductive surgery is achieved. The challenge is the correct patient selection for secondary cytoreductive surgery to improve the results of this approach.

## 1. Introduction

Almost 75% of new high-grade epithelial ovarian cancer diagnoses debut at an advanced stage. In this scenario, complete cytoreduction surgery is the cornerstone of management in primary treatment [1].

About 80% of patients will relapse despite chemotherapy treatment (CHT) and targeted maintenance therapy [2]. The treatment of recurrent ovarian cancer (ROC) is thus a significant clinical challenge [3].

However, the use of proteomics and the presence of mutation detection are currently being developed in advanced ovarian cancer in order to outline and establish molecular subgroups that, in the future, will allow the use of therapeutic targets, improving the prognosis of our patients [4,5].

The standard treatment for ROC patients includes intravenous CHT, chosen based on platinum sensitivity [4]. It is important to clarify the role of secondary cytoreductive surgery (SCS) in these patients and the choice of a surgical approach [1,2,5,6,7,8,9,10,11,12].

Numerous retrospective studies have shown that SCS improves overall survival (OS) in platinum-sensitive recurrent patients compared to chemotherapy alone [2] and that complete resection is the most important prognostic factor, as in the primary treatment of ovarian cancer [6,7,8].

Due to the fact that these studies are retrospective and too heterogeneous, they do not allow reliable conclusions [1,2,5,9,10,13,14].

Therefore, the results obtained in reference to disease-free survival (DFS) and OS associated with SCS + CHT compared to CHT present little scientific evidence [2].

Bizarri et al. demonstrated that in patients with neoadjuvant chemotherapy and interval surgery, there was no difference in progression-free survival [1], data that were extrapolated to patients with relapse [2,15].

The theoretical benefit of SCS in recurrence ovarian cancer is based on enhancement of the chemotherapeutic effect by removing large tumor volumes with a poor blood supply, equal to that of primary surgery in advanced ovarian cancer [5]. This is the reason why complete resection is the main prognostic factor in primary cytoreductive surgery in ovarian cancer, and it is possible to extrapolate this benefit to the SCS. It is a challenge to select the patients who will benefit from the procedure in primary and recurrent surgery [10].

A Cochrane meta-analysis shows a better survival outcome in ROC patients when the absence of tumor residue is achieved, particularly in platinum-sensitive patients [16]. To achieve this complete cytoreduction, predictive models must be used to allow the correct patient selection [16,17].

In DESKTOP II, a selection model for SCS is proposed based on the absence of ascites at recurrence, an Eastern cooperative oncology group (ECOG) performance status of 0, and no tumor residue at primary surgery [6]. In the study of Tian et al., the proposed selection model is based on the previous criteria in addition to FIGO stage and Ca 125 value [1].

Due to the lack of scientific evidence with sufficient statistical power during the 2010s, three randomized articles were designed to assess whether SCS + CHT has better prognostic outcomes than CHT alone in patients with recurrent ovarian cancer.

It should be noted that some retrospective studies do not consider the use of maintenance therapy with iPARPs or anti-VEGF, which is why the information obtained from randomized studies in this field with a 5-year follow-up allows for highly reliable results compared to those previously published.

The purpose of this systematic review and meta-analysis is to evaluate the prognostic impact of SCS and CHT on patients with ROC (OS and DFS). Additionally, we perform a subgroup analysis to assess the impact of complete cytoreduction in these patients.

## 2. Methods

### 2.1. Search Strategy

This analysis was reported in accordance with the preferred reporting items for systematic reviews and meta-analyses (PRISMA) guidelines (PRISMA 2020, http://www.prismastatement.org).

From 2017 to April 2022, the PubMed and Cochrane central register of controlled trials databases were systematically searched to compare the results of secondary cytoreduction surgery plus chemotherapy with the use of chemotherapy alone in recurrent ovarian cancer.

The search terms used for all databases were (‘Relapse ovarian cancer’ OR ‘recurrent ovarian cancer’) AND (‘surgery’), and randomized controlled clinical trials were included. The information extracted from each article chosen according to the inclusion criteria was the following: study design, study population, median or average age, intervention method, median OS, and median DFS. The hazard ratio of the intervention measure and the 95% confidence interval (CI) for OS and DFS were obtained for each article.

Currently, following the recommendations of the Vancouver consensus, optimal surgery is defined as surgery without macroscopic evidence of disease after surgery (R0) and suboptimal with evidence of a residual tumor (R1-2) [16]. In the included articles, the term incomplete surgery is used for any amount of tumor residue, and complete surgery is used to indicate no macroscopic residual disease.

### 2.2. Inclusion and Exclusion Criteria

According to the PICOS criteria (population, intervention, comparison, outcome, and study design), randomized clinical studies in patients with relapsed ovarian cancer comparing two treatment groups—chemotherapy alone vs. surgery plus chemotherapy—and assessing DFS and OS outcomes were selected. We included studies published in the last 5 years in the Spanish and English languages and excluded articles lacking complete cytoreduction data and with the presence of extra-abdominal disease.

### 2.3. Selection Process

Two review authors independently evaluated the eligibility of the papers and resolved their disagreements by discussion or by appeal to a third review author.

The search was performed using the following filters:

- Languages: English and Spanish
- Date: the last 5 years
- Type of study: randomized controlled trial

### 2.4. Statistical Analysis

The objective of the study is the assessment of OS and DFS using the association measure hazard ratio through the model fixed effect regardless of the heterogeneity due to the small number of articles included in the statistical analysis. The results obtained are represented using the forest plot graph. The results are considered statistically significant when the *p* > 0.05.

The heterogeneity of the articles included is assessed using the I2 tool, with a value of less than 25% being considered low heterogeneity, 50% intermediate, and greater than or equal to 75% being considered high heterogeneity.

All the results were evaluated using RevMan 5 software (Review Manager [RevMan] Version 5.4, The Cochrane Collaboration, 2020).

## 3. Results

### 3.1. Selected Studies

**Figure 1:**
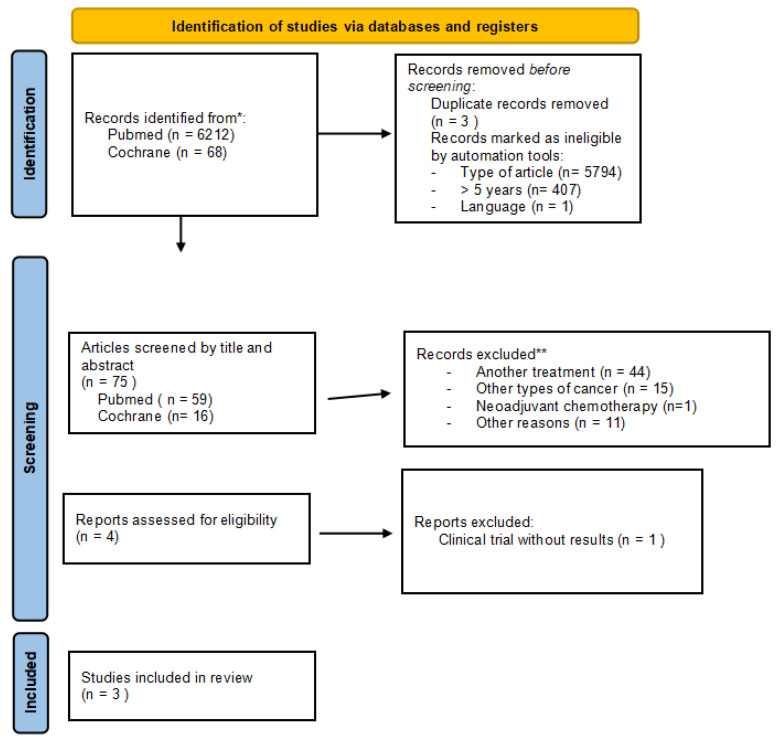
PRISMA diagram showing the selection process of the articles included in the study.

The search strategy uncovered a total of 6212 results in PubMed Research and 68 in Cochrane Research. After filtering articles with electronic tools and eliminating duplicates, 3 articles in PubMed and 1 article in Cochrane were selected. Finally, three articles were included in the review. Figure 1 shows the selection process carried out and the reasons for the exclusion of the articles.

**Figure 2:**
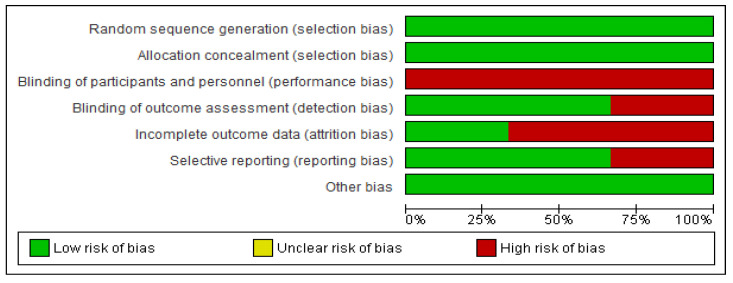
Risk of bias summary.

**Figure 3:**
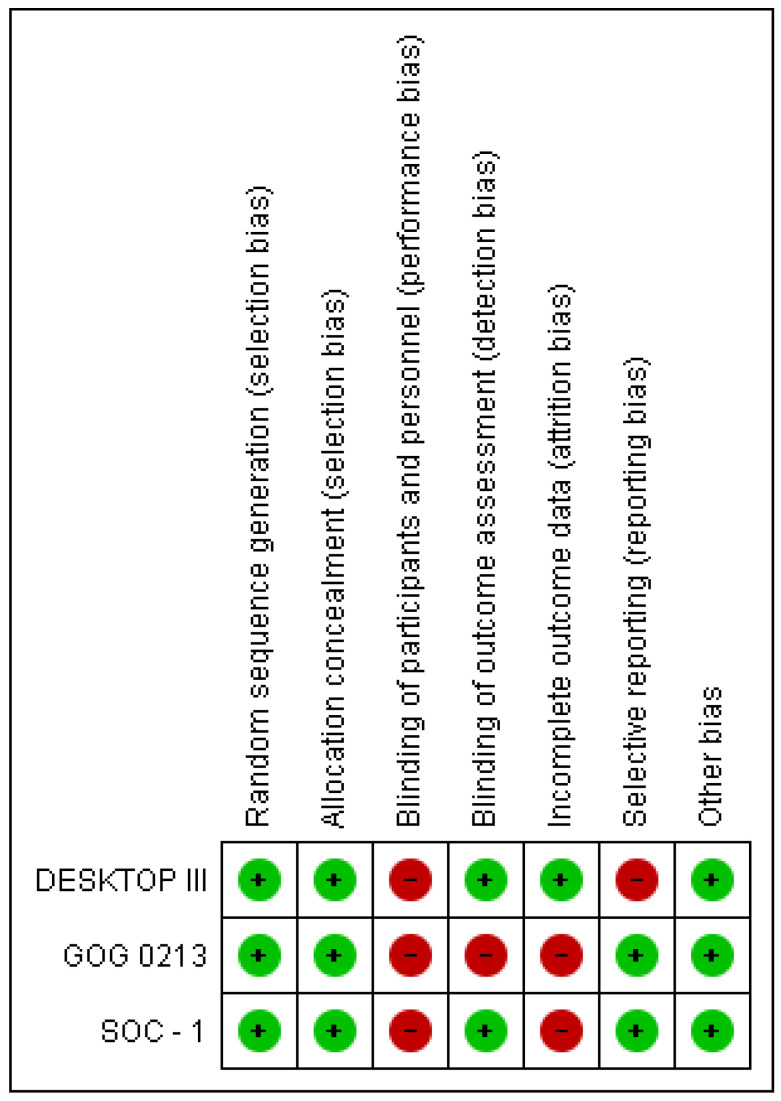
Risk of bias graph.

The three randomized clinical trials (RCTs) selected included a study population of 1249 patients. Table 1 and Table 2 show highlights of the included studies. Bias analyses are detailed in Figure 2 and Figure 3.

### 3.2. Overall Meta-Analyses of OS and DFS

The three articles showed OS and DFS. The results obtained from each study are represented in Table 3.

**Figure 4:**
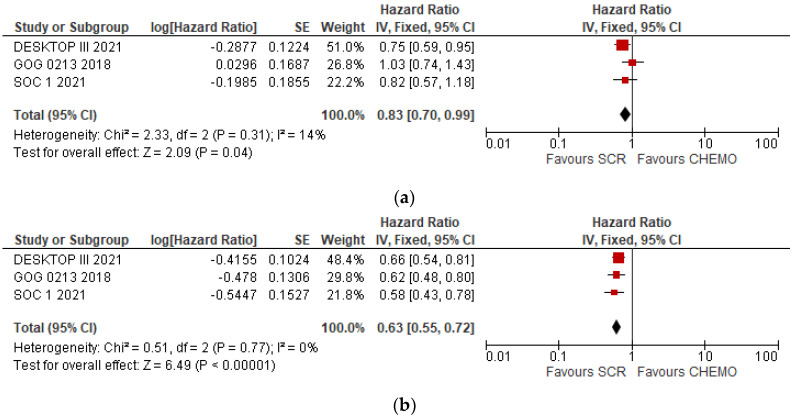

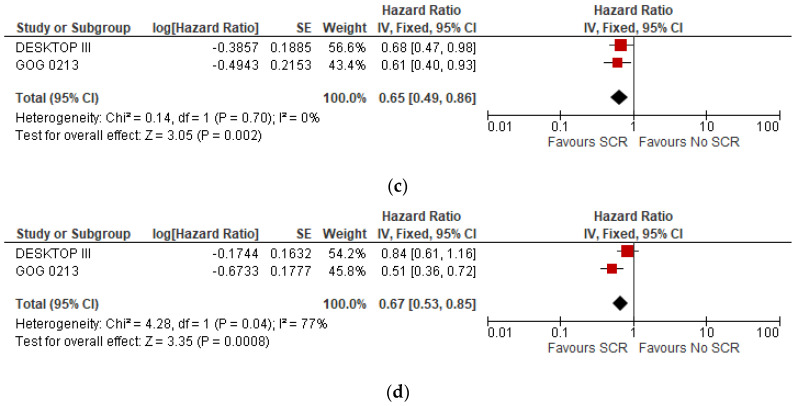
(**a**): Statistical study OS: Results obtained from all studies included in the review. (**b**): Statistical study DFS: Results obtained from all studies included in the review. (**c**): Subgroup analysis of overall survival with complete cytoreductive surgery vs. residual tumor. (**d**): Subgroup analysis of disease-free survival with complete cytoreductive surgery vs. residual tumor.

Analysis of the data obtained exhibited an improvement in OS in the secondary cytoreductive surgery group of patients compared to standard chemotherapy treatment. (HR 0.83, IC 95% 0.70–0.99, *p* = 0.04) (Figure 4a).

Additionally, the DFS analysis showed statistically significant differences between two groups, with an improvement in the group of patients who underwent CRS (Figure 4b) (HR 0.63, IC 95% 0.55–0.72, *p* < 0.000001).

### 3.3. Subgroup Analysis

OS and DFS results were collected and analysed according to whether complete resection was achieved. Only two studies showed relevant results. These studies suggested a statistically significant improvement in both OS (HR = 0.65, IC 95% 0.49–0.86, *p* = 0.002) and DFS (HR = 0.67, IC 95% 0.36–0.72, *p* = 0.0008) when complete resection was achieved (Figure 4c,d).

## 4. Discussion

The principal findings of our meta-analysis suggest that in patients with ROC, SCS plus chemotherapy could improve OS and DFS, and this benefit is greater in those patients without tumor residue.

The results of DESKTOP III and SOC-1 showed an improvement in overall survival and disease-free interval in the first two studies, while GOG 0213 only shows a benefit in disease-free interval in the complete resection surgery population.

It is necessary to determine whether this benefit is intrinsic to the surgery or whether it derives from the selection of patients with a better prognosis [18,19]. In addition to determining the benefit derived from surgery that offsets those secondary to the economic cost of these highly complex surgeries, hospital stays, complications, and possible delays in the initiation of chemotherapy treatment.

Moreover, it is necessary to determine the patient groups most likely to benefit from this treatment. [16,18,20]

The ability to select ideal candidates for secondary surgery represents a clinical challenge, as has already been seen in primary cytoreductive surgery [1,3,4,9,13].

Two of the articles analyzed used methods for selecting patients for secondary cytoreduction. Coincidentally, the same two articles showed the best results for OS and DFS in the SCS group (DESKTOP III and SOC-1) [19,21]., In the GOG 0213 study, the selection criterion was the surgeon’s decision; these differences in OS between the two groups were not evident, but a better prognosis was observed in the chemotherapy group [7]. However, better results for DFS were evidenced in the SCS group. These are the data used in the statistical analysis because the complete debulking rate in this article was the lowest of the three, there was an unspecified percentage of cross-over between the non-surgical and surgical groups that could benefit the chemotherapy group alone, and patient selection was based on surgeon judgment. Therefore, to minimize heterogeneity, it was decided to use this hazard ratio value.

The method of patient selection was an important difference between the studies. The DESKTOP-III trial used the selection method described in DESKTOP-I (AGO score) [6]. This method is based on presenting a performance status of 0 according to the Eastern cooperative oncology group (ECOG) scale, the presence of ascites at recurrence, and complete resection at primary surgery. This algorithm has a predicted rate of complete cytoreduction in patients with recurrence of more than 66% [4,9,19].

By contrast, in the SOC-1 article, the algorithm is based on the following clinical data: FIGO stage at diagnosis, presence of residual tumor at primary surgery, platinum sensitivity interval, performance status (ECOG), Ca 125 level at relapse, and the presence of ascites at recurrence, with the addition of PET CT findings [1,21].

The selection criteria in the models used in DESKTOP III and SOC-1 are different, so the selected population differs between them. The iMODEL in SOC-1 selected more candidates; moreover, these patients are younger compared with those enrolled in DESKTOP III and GOG 0213 [22,23,24]. That fact could explain the improvement in results and the higher selection of patients for the experimental group in SOC-1. However, the platinum-free interval in the SOC-1 study is the shortest of all, being 16 months, compared to 20.4 months in GOG 0213 and 21.1 in DESKTOP III, which could translate into a worse prognosis profile.

The DESKTOP-III and SOC-1 trials led to complete resection in 74.5% and 77% of the patients, respectively, higher percentages than obtained in the GOG-0213 trial (67%) [1,5,19,24]. Due to the aforementioned, it seems that an objective model for selecting patients who are candidates for SCS could be the reason for the higher rates of complete cytoreduction and evidence of impact on OS and DFS in the SCS group in both trials (DESKTOP-III and SOC-1), in contrast to the GOG-0213 trial.

Another factor to consider in the selection of patients for SCS is that it is based on patient selection models that do not include laparoscopic assessment. Llueca et al. show that for proper selection of patients for primary surgery, the addition of diagnostic laparoscopy increases the accuracy of patient selection [18,22,23,24]. Similarly, Gallota et al. show that the use of laparoscopy in selected patients with ROC allows cytoreduction to be performed in addition to assessment of the extent of disease [14,25]. It is possible that the addition of diagnostic laparoscopy in the previous selection model increased the number of patients with complete resection.

Another difference is the use of adjuvant therapy in three articles, specifically the use of bevacizumab. The activity of bevacizumab is not comparable to the beneficial effect of surgery in patients with recurrence [5,26,27]. Bevacizumab was used in 84% of patients in the GOG-0213 trial, while it was only used in 23% and 1% of patients in DESKTOP III and SOC-1, respectively, in the non-surgical chemotherapy group. It should be noted that the latter two trials were mainly surgical, while GOG-0213 performed a mixture of chemotherapy and surgery in patients with ROC.

To assess the effect of bevacizumab, GOG 0213 conducted an analysis in the group of patients who had not received maintenance bevacizumab. This subgroup showed persistent improvement in the benefit of chemotherapy over SCR regardless of bevacizumab use, with an OS of 67 months in the first group compared to 32.4 months in the SCR group [2,9,24].

However, it should be noted that these results were obtained in a subgroup of patients that corresponds to less than 20% of the total number of patients included, this subgroup not having been defined prospectively and before the article was written [7,9].

Moreover, only 6% of patients enrolled had not received bevacizumab at the time of randomization. A detriment in OS was observed in the surgery group that did not receive bevacizumab (HR: 2.29, 95% IC: 1.31–3.88); meanwhile, this difference was not that evident in the surgery and no surgery groups plus bevacizumab: 61.7 months *vs.* 58.5 months, respectively. It is possible that the non-use of bevacizumab makes subsequent lines of therapy less likely to affect long-term outcomes [26,27].

Although the subgroup of patients who did not receive bevacizumab in the DESKTOP III and in the SOC-1 trials (77% and 99% of the trial population, respectively) was larger than that in the GOG-0213 trial, the analysis of this subgroup was neither blind nor placebo-controlled, and the analysis of this subgroup was not established in the initial study design, which created a substantial bias, and the results are of low confidence [5,19,24].

Chemotherapy treatment after CRS is not standardized and was different in the included studies.

It is possible that the choice of one treatment or another is influenced by different factors, such as postoperative complications, randomization outcome, residual tumor, or the preferences of patients or investigators.

Therefore, it is not possible to draw conclusions about the benefit of bevacizumab in patients with incomplete surgery or whether the effect of bevacizumab is comparable to the benefit of surgery in relapsed ovarian cancer [23].

Another key point is that the overall survival of patients in the chemotherapy group in GOG 0213 is longer than expected in that population group, at 64.7 months, based on the results of published retrospective studies [7].

The OS obtained in GOG 0213 in the SCR group is 56 months, with no statistically significant differences compared to the chemotherapy group [7,9]. This decrease in survival could be a consequence of the delay in starting chemotherapy after surgery. In SOC-1, the period from surgery to the start of chemotherapy was 16 days, while in GOG 0213 it was 40 days. In this same study, a post hoc study was performed showing an increased risk of death after 25 days with a delay in chemotherapy [7]. This worsening in survival due to the delay of chemotherapy had already been demonstrated in retrospective studies.

Another limitation of the included studies is the cross-over of patients from the standard treatment group to the SCR group, which in SOC-1 reaches up to 37% of patients. This fact is a selection bias, decreasing the statistical power of the study to detect a negative result in OS in the control group [24].

All three studies are multicenter studies, and it is convenient to highlight the need to perform complete cytoreduction surgeries in reference centers to ensure excellence in the procedure [28,29,30]. In DESKTOP-III and SOC-1, it is specified in the material and methods that the centers chosen to be included in the study were selected according to the number of procedures they had performed on patients with advanced ovarian cancer and previous experience in studies in ovarian cancer, which possibly also influenced the rates of complete cytoreduction. GOG 0213 does not specify the method of selection used by the centers included in the study.

In the present meta-analysis, there are a few limitations, as we have observed during the discussion. First, several criteria were used to select patients for randomization, which could lead to selective bias. Second is the absence of standardization of complementary treatment after surgery (addition of bevacizumab or iPARP to conventional chemotherapy).

There are other factors to consider in the ROC, such as tumor histology, somatic or germline mutation, homologous recombination deficiency (HRD) or BRCA status, previous use or relapse during use of antiangiogenic drug (bevacizumab) or PARP inhibitor (PARPi) treatment, and the pattern of relapse presentation [13,26,27,31,32,33], which could change the management of the ROC [33,34,35,36]. Some of these treatments have shown a DFS benefit among patients with ROC.

Moreover, it has been shown that in a certain group of patients with BRCA1/BRCA2 wild type, they present an immune profile of the tumor showing the expression of PD-1/PDL-1 that would benefit from the treatment of iPARP associated with immunotherapy directed at these two biomarkers. That is why, currently, the genomic analysis of the tumor is a fundamental step in the treatment of patients with ovarian cancer [35,37,38].

A weakness of the included studies is that none of them specify the pattern of relapse or the mutational status of BRCA. This could imply a selection bias because the somatic or germline mutation in BRCA 1/2 confers chemosensitivity. In addition, BRCA status plays a role in the treatment of patients with relapse [38,39]. BRCA mutational status appears to determine the possibility of secondary cytoreduction in patients with liver metastases showing better PFS, which is relevant information to know in patients with ovarian cancer [38].

The principal strength of this meta-analysis is that it is the only study that includes the final results of three RCTs, which gives the results of this study the highest level of evidence to date in the surgical treatment of ROC. Another strength of this study is that the low percentage of heterogeneity obtained when comparing the RCTs offers a high level of evidence for the results of the meta-analysis.

The results of this review show an improvement in OS and DFS in those patients who underwent SCR. This benefit is increased in those in which complete cytoreduction is achieved.

These findings are consistent with the benefit of chemotherapy in patients with complete debulking in primary surgery due to the hypothesized potential effect of chemotherapy in patients without residual tumors.

## 5. Conclusions

In conclusion, SCR might result in improved DFS and OS in patients with ROC. Complete cytoreductive surgery could bring survival benefits. The achievement of complete surgery highlights the need for the development of a selection model for secondary cytoreductive surgery. These findings redefine a new standard of care for patients with recurrent, platinum-sensitive ovarian cancer. However, it is worth considering the possibility of a change in the recurrence type in patients on targeted maintenance therapy, as platinum sensitivity classification may not be sufficient to determine whether a patient is amenable to surgery for ovarian cancer relapse.

## Figures and Tables

**Table 1:** Principal results of included randomized clinical trials.

	Groups	N of Patients	Selection Criteria	CTC %	Survival In Scs	Survival in No Scs
**DESKTOP III**	SCS No SCS	206 201	AGO score	76%	53.7 m (OS)	46 m (OS)
**GOG 0213**	SCS No SCS	240 245	None	67%	50.6 m (OS)	64.7 m (OS)
**SOC-1**	SCS No SCS	182 175	IModel + PET TC	77%	58.1 m (OS)	53.9 m (OS)

CTC, complete tumor cytoreduction; PFS, progression-free survival; OS, overall survival; SCS, secondary cytoreductive surgery; No SCS, chemotherapy alone; PET TC, positron emission tomography—computed tomography.

**Table 2:** Characteristics of patients in randomized clinical trials.

	Age	FIGO	Platinum-Free Interval	Previous Bevacizumab	Histology
**DESKTOP III**	SCS 60.8 (54.2–67.3)	III 145 (70.4%) IV 16 (7.8%)	6–12 m 48 (23.6%) >12 m 155 (76.4%)	33 (16%)	Serous 177 (85.9%) Other 23 (14.1%)
	No SCS 62.2 (54.2–69.9)	III 143 (71.1%) IV 13 (6.5%)	6–12 m 47 (23.7%) >12 m 151 (76.3%)	31 (15.4%)	Serous 161 (80.1%) Other 40 (19.9%)
**GOG 0213**	SCS <60 years 135 (56.2%) >60 years 105 (43.8%)	NE	NE	25 (10.4%)	Serous 211 (87%) Other 29 (12.3%)
	No SCS <60 years 145 (59.1%) >60 years 100 (40.8%)			30 (12.2%)	Serous 207 (84.5%) Other 38 (15.5%)
**SOC-1**	SCS <54 years 80 >54 years 102	III 128 IV 20	NE	NE	Serous 158 Another 24
	No SCS <54 years 90 >54 years 85	III 121 IV 25			Serous 145 Another 30

**Table 3:** Main results of the included articles.

	Groups	DFS (Median Months)	HR	IC 95%	*P*	OS (Median Months)	HR	IC 95%	*P*
**DESKTOP III**	SCS	18.4	0.66	12.7–20.8	NE	53.7	0.75	0.59–0.96	0.02
	No SCS	14				46			
**GOG 0213**	SCS	18.9	0.62	0.48–0.80	NE	50.6	1.03	0.74–1.43	0.08
	No SCS	16.2				64.7			
**SOC-1**	SCS	17.4	0.58	0.45–0.74	<0.0001	58.1	0.82	0.57–1.19	NE
	No SCS	11.9				53.9			

## Data Availability

Data sharing is not applicable to this article as no new data were created or analyzed in this study.

## References

[1] Tian W.J., Chi D.S., Sehouli J., Tropé C.G., Jiang R., Ayhan A., Cormio G., Xing Y., Breitbach G.P., Braicu E.I. (2012). A risk model for secondary cytoreductive surgery in recurrent ovarian cancer: An evidence-based proposal for patient selection. Ann. Surg. Oncol..

[2] Ding T., Tang D., Xi M. (2021). The survival outcome and complication of secondary cytoreductive surgery plus chemotherapy in recurrent ovarian cancer: A systematic review and meta-analysis. J. Ovarian Res..

[3] Winter W.E., Maxwell G.L., Tian C., Carlson J.W., Ozols R.F., Rose P.G., Markman M., Armstrong D.K., Muggia F., McGuire W.P. (2007). Prognostic Factors for Stage III Epithelial Ovarian Cancer: A Gynecologic Oncology Group Study. J. Clin. Oncol..

[4] Wood-Bouwens C.M., Haslem D., Moulton B., Almeda A.F., Lee H., Heestand G.M., Nadauld L.D., Ji H.P. (2019). Therapeutic Monitoring of Circulating DNA Mutations in Metastatic Cancer with Personalized Digital PCR. J. Mol. Diagn..

[5] Wan J.C.M., Mughal T.I., Razavi P., Dawson S.-J., Moss E.L., Govindan R., Tan I.B., Yap Y.-S., Robinson W.A., Morris C.D. (2021). Liquid biopsies for residual disease and recurrence. Med.

[6] Harter P., Du Bois A., Hahmann M., Hasenburg A., Burges A., Loibl S., Gropp M., Huober J., Fink D., Schröder W. (2006). Surgery in Recurrent Ovarian Cancer: The Arbeitsgemeinschaft Gynaekologische Onkologie (AGO) DESKTOP OVAR Trial. Ann. Surg. Oncol..

[7] Coleman R.L., Spirtos N.M., Enserro D., Herzog T.J., Sabbatini P., Armstrong D.K., Kim J.-W., Park S.-Y., Kim B.-G., Nam J.-H. (2019). Secondary Surgical Cytoreduction for Recurrent Ovarian Cancer. N. Engl. J. Med..

[8] Shi T., Yin S., Zhu J., Zhang P., Liu J., Xiang L., Zhu Y., Wu S., Chen X., Wang X. (2020). A phase II trial of cytoreductive surgery combined with niraparib maintenance in platinum-sensitive, secondary recurrent ovarian cancer: SGOG SOC-3 study. J. Gynecol. Oncol..

[9] Conte C., Fagotti A., Avesani G., Trombadori C., Federico A., D’indinosante M., Giudice M.T., Pelligra S., Lodoli C., Marchetti C. (2021). Update on the secondary cytoreduction in platinum-sensitive recurrent ovarian cancer: A narrative review. Ann. Transl. Med..

[10] Al Rawahi T., Lopes A.D., E. Bristow R., Bryant A., Elattar A., Chattopadhyay S., Galaal K. (2013). Surgical cytoreduction for recurrent epithelial ovarian cancer. Cochrane Database Syst. Rev..

[11] Harter P., Sehouli J., Reuss A., Hasenburg A., Scambia G., Cibula D., Mahner S., Vergote I., Reinthaller A., Burges A. (2011). Prospective Validation Study of a Predictive Score for Operability of Recurrent Ovarian Cancer: The Multicenter Intergroup Study DESKTOP II. A Project of the AGO Kommission OVAR, AGO Study Group, NOGGO, AGO-Austria, and MITO. Int. J. Gynecol. Cancer.

[12] Lee C.K., Lord S., Grunewald T., Gebski V., Hardy-Bessard A.-C., Sehouli J., Woie K., Heywood M., Schauer C., Vergote I. (2014). Impact of secondary cytoreductive surgery on survival in patients with platinum sensitive recurrent ovarian cancer: Analysis of the CALYPSO trial. Gynecol. Oncol..

[13] Zang R.Y., Harter P., Chi D.S., Sehouli J., Jiang R., Tropé C.G., Ayhan A., Cormio G., Xing Y., Wollschlaeger K.M. (2011). Predictors of survival in patients with recurrent ovarian cancer undergoing secondary cytoreductive surgery based on the pooled analysis of an international collaborative cohort. Br. J. Cancer.

[14] Gallotta V., Conte C., Giudice M.T., Nero C., Vizzielli G., Alletti S.G., Cianci S., Lodoli C., Di Giorgio A., De Rose A.M. (2018). Secondary Laparoscopic Cytoreduction in Recurrent Ovarian Cancer: A Large, Single-Institution Experience. J. Minim. Invasive Gynecol..

[15] Bizzarri N., Marchetti C., Conte C., Loverro M., Giudice M.T., Quagliozzi L., Distefano M., Chiantera V., Scambia G., Fagotti A. (2022). The impact of secondary cytoreductive surgery in platinum sensitive recurrent ovarian cancer treated with upfront neoadjuvant chemotherapy and interval debulking surgery. Gynecol. Oncol..

[16] Kim S.I., Cho J., Lee E.J., Park S., Park S.J., Seol A., Lee N., Yim G.W., Lee M., Lim W. (2019). Selection of patients with ovarian cancer who may show survival benefit from hyperthermic intraperitoneal chemotherapy: A systematic review and meta-analysis. Medicine (United States)..

[17] Matsumoto A., Higuchi T., Yura S., Mandai M., Kariya M., Takakura K., Fujii S. (2006). Role of salvage cytoreductive surgery in the treatment of patients with recurrent ovarian cancer after platinum-based chemotherapy. J. Obstet. Gynaecol. Res..

[18] Vergote I., Tropé C.G., Amant F., Kristensen G.B., Ehlen T., Johnson N., Verheijen R.H.M., van der Burg M.E.L., Lacave A.J., Panici P.B. (2010). Neoadjuvant chemotherapy or primary surgery in stage IIIC or IV ovarian cancer. N. Engl. J. Med..

[19] Fagotti A., Ferrandina M.G., Vizzielli G., Pasciuto T., Fanfani F., Gallotta V., Margariti P.A., Chiantera V., Costantini B., Alletti S.G. (2020). Randomized trial of primary debulking surgery versus neoadjuvant chemotherapy for advanced epithelial ovarian cancer (SCORPION-NCT01461850). Int. J. Gynecol. Cancer.

[20] Stuart G.C., Kitchener H., Bacon M., Dubois A., Friedlander M., Ledermann J., Marth C., Thigpen T., Trimble E. (2011). 2010 Gynecologic Cancer InterGroup (GCIG) Consensus Statement on Clinical Trials in Ovarian Cancer: Report From the Fourth Ovarian Cancer Consensus Conference. Int. J. Gynecol. Cancer.

[21] Llueca A., Serra A., Delgado K., Maiocchi K., Jativa R., Gomez L., Escrig J. (2019). A radiologic-laparoscopic model to predict suboptimal (or complete and optimal) debulking surgery in advanced ovarian cancer: A pilot study. Int. J. Women’s Heal..

[22] Llueca A., Serra A., Rivadulla I., Gomez L., Escrig J., MUAPOS working group (Multidisciplinary Unit of Abdominal Pelvic Oncology Surgery) (2018). Prediction of suboptimal cytoreductive surgery in patients with advanced ovarian cancer based on preoperative and intraoperative determination of the peritoneal carcinomatosis index. World J. Surg. Oncol..

[23] Harter P., Sehouli J., Vergote I., Ferron G., Reuss A., Meier W., Greggi S., Mosgaard B.J., Selle F., Guyon F. (2021). Randomized Trial of Cytoreductive Surgery for Relapsed Ovarian Cancer. New Engl. J. Med..

[24] Shi T., Zhu J., Feng Y., Tu D., Zhang Y., Zhang P., Jia H., Huang X., Cai Y., Yin S. (2021). Secondary cytoreduction followed by chemotherapy versus chemotherapy alone in platinum-sensitive relapsed ovarian cancer (SOC-1): A multicentre, open-label, randomised, phase 3 trial. Lancet Oncol..

[25] Fagotti A., Fanfani F., Vizzielli G., Gallotta V., Ercoli A., Paglia A., Costantini B., Vigliotta M., Scambia G., Ferrandina G. (2010). Should laparoscopy be included in the work-up of advanced ovarian cancer patients attempting interval debulking surgery?. Gynecol. Oncol..

[26] Petrillo M., Amadio G., Salutari V., Paris I., Di Stefano M., Ferandina G., Scambia G., Fagotti A. (2016). Impact of bevacizumab containing first line chemotherapy on recurrent disease in epithelial ovarian cancer: A case-control study. Gynecol. Oncol..

[27] Coleman R.L., Brady M.F., Herzog T.J., Sabbatini P., Armstrong D.K., Walker J.L., Kim B.G., Fujiwara K., Tewari K.S., O’Malley D.M. (2017). Bevacizumab and paclitaxel&ndash;carboplatin chemotherapy and secondary cytoreduction in recurrent, platinum-sensitive ovarian cancer (NRG Oncology/Gynecologic Oncology Group study GOG-0213): A multicentre, open-label, randomised, phase 3 trial. Lancet Oncol..

[28] Llueca A., Serra A., Climent M.T., Segarra B., Maazouzi Y., Soriano M., Escrig J., on behalf MUAPOS Working Group (2020). Outcome quality standards in advanced ovarian cancer surgery. World J. Surg. Oncol..

[29] Llueca A., Climent M.T., Escrig J., Carrasco P., Serra A., Gomez-Quiles L., Játiva R., Cebrian G., Bosso V., Villarin A. (2021). Validation of three predictive models for suboptimal cytoreductive surgery in advanced ovarian cancer. Sci. Rep..

[30] Fotopoulou C., Concin N., Planchamp F., Morice P., Vergote I., Du Bois A., Querleu D. (2020). Quality indicators for advanced ovarian cancer surgery from the European Society of Gynaecological Oncology (ESGO): 2020 update. Int. J. Gynecol. Cancer.

[31] Oza A.M., Cibula D., Benzaquen A.O., Poole C., Mathijssen R.H., Sonke G., Colombo N., Špaček J., Vuylsteke P., Hirte H. (2015). Olaparib combined with chemotherapy for recurrent platinum-sensitive ovarian cancer: A randomised phase 2 trial. Lancet Oncol..

[32] Pujade-Lauraine E., Ledermann J.A., Selle F., Gebski V., Penson R.T., Oza A.M., Korach J., Huzarski T., Poveda A., Pignata S. (2017). Olaparib tablets as maintenance therapy in patients with platinum-sensitive, relapsed ovarian cancer and a BRCA1/2 mutation (SOLO2/ENGOT-Ov21): A double-blind, randomised, placebo-controlled, phase 3 trial. Lancet Oncol..

[33] Revythis A., Limbu A., Mikropoulos C., Ghose A., Sanchez E., Sheriff M., Boussios S. (2022). Recent Insights into PARP and Immuno-Checkpoint Inhibitors in Epithelial Ovarian Cancer. Int. J. Environ. Res. Public Heal..

[34] González-Martín A., Pothuri B., Vergote I., DePont Christensen R., Graybill W., Mirza M.R., McCormick C., Lorusso D., Hoskins P., Freyer G. (2019). Niraparib in Patients with Newly Diagnosed Advanced Ovarian Cancer. N. Engl. J. Med..

[35] Boussios S., Rassy E., Moschetta M., Ghose A., Adeleke S., Sanchez E., Sheriff M., Chargari C., Pavlidis N. (2022). BRCA Mutations in Ovarian and Prostate Cancer: Bench to Bedside. Cancers.

[36] Boussios S., Moschetta M., Karihtala P., Samartzis E.P., Sheriff M., Pappas-Gogos G., Ozturk M.A., Uccello M., Karathanasi A., Tringos M. (2020). Development of new poly(ADP-ribose) polymerase (PARP) inhibitors in ovarian cancer: Quo Vadis?. Ann Transl. Med..

[37] Gallotta V., Bruno M., Conte C., Giudice M.T., Davià F., Moro F., Zannoni G.F., Fagotti A., De Bonis M., Capoluongo E. (2020). Salvage lymphadenectomy in recurrent ovarian cancer patients: Analysis of clinical outcome and BRCA1/2 gene mutational status. Eur. J. Surg. Oncol..

[38] Gallotta V., Conte C., D’indinosante M., Capoluongo E., Minucci A., De Rose A.M., Ardito F., Giuliante F., Di Giorgio A., Zannoni G.F. (2019). Prognostic factors value of germline and somatic brca in patients undergoing surgery for recurrent ovarian cancer with liver metastases. Eur. J. Surg. Oncol. (EJSO).

[39] Hollis R.L., Churchman M., Gourley C. (2017). Distinct implications of different BRCA mutations: Efficacy of cytotoxic chemotherapy, PARP inhibition and clinical outcome in ovarian cancer. OncoTargets Ther..

